# Di-μ-hydroxido-bis­[aqua­trichlorido­tin(IV)] diethyl ether disolvate

**DOI:** 10.1107/S1600536808032832

**Published:** 2008-10-18

**Authors:** Minglei Yang, Handong Yin, Li Quan, Liansheng Cui, Daqi Wang

**Affiliations:** a College of Chemistry and Chemical Engineering, Liaocheng University, Shandong 252059, People’s Republic of China

## Abstract

The title compound, [Sn_2_Cl_6_(OH)_2_(H_2_O)_2_]·2C_4_H_10_O, consists of a centrosymmetric molecule and two additional solvent molecules and has an infinite two-dimensional network extending parallel to (101). The Sn atom is six-coordinate with a distorted octa­hedral geometry. Additional O—H⋯O hydrogen bonding leads to stabilization of the crystal structure.

## Related literature

For a related structure, see: Janas *et al.* (1991 ▶)
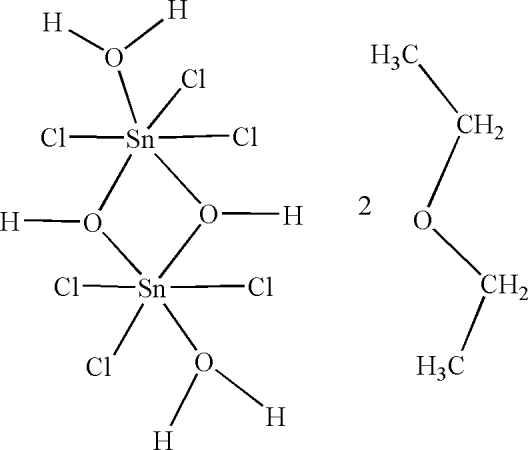


## Experimental

### 

#### Crystal data

[Sn_2_Cl_6_(OH)_2_(H_2_O)_2_]·2C_4_H_10_O
*M* *_r_* = 668.36
Monoclinic, 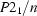

*a* = 10.1171 (15) Å
*b* = 10.0212 (15) Å
*c* = 11.2641 (18) Å
β = 103.536 (1)°
*V* = 1110.3 (3) Å^3^
*Z* = 2
Mo *K*α radiation
μ = 2.99 mm^−1^
*T* = 298 (2) K
0.46 × 0.32 × 0.30 mm

#### Data collection

Siemens SMART CCD area-detector diffractometer
Absorption correction: multi-scan (*SADABS*; Sheldrick, 1996 ▶) *T*_min_ = 0.340, *T*_max_ = 0.468 (expected range = 0.297–0.408)
5168 measured reflections
1909 independent reflections
1685 reflections with *I* > 2σ(*I*)
*R*_int_ = 0.027

#### Refinement

*R*[*F* ^2^ > 2σ(*F* ^2^)] = 0.023
*wR*(*F* ^2^) = 0.063
*S* = 0.84
1909 reflections
102 parameters
H-atom parameters constrained
Δρ_max_ = 1.05 e Å^−3^
Δρ_min_ = −0.68 e Å^−3^

### 

Data collection: *SMART* (Siemens, 1996 ▶); cell refinement: *SAINT* (Siemens, 1996 ▶); data reduction: *SAINT*; program(s) used to solve structure: *SHELXS97* (Sheldrick, 2008 ▶); program(s) used to refine structure: *SHELXL97* (Sheldrick, 2008 ▶); molecular graphics: *SHELXTL* (Sheldrick, 2008 ▶); software used to prepare material for publication: *SHELXTL*.

## Supplementary Material

Crystal structure: contains datablocks I, global. DOI: 10.1107/S1600536808032832/sg2266sup1.cif

Structure factors: contains datablocks I. DOI: 10.1107/S1600536808032832/sg2266Isup2.hkl

Additional supplementary materials:  crystallographic information; 3D view; checkCIF report

## Figures and Tables

**Table 1:** Hydrogen-bond geometry (Å, °)

*D*—H⋯*A*	*D*—H	H⋯*A*	*D*⋯*A*	*D*—H⋯*A*
O1—H1⋯O3^i^	0.93	1.88	2.799 (3)	169
O2—H2*D*⋯O3^ii^	0.85	1.89	2.736 (3)	176

Symmetry codes: (i) 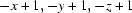; (ii) 
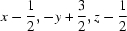
.

**Fig. 1.**
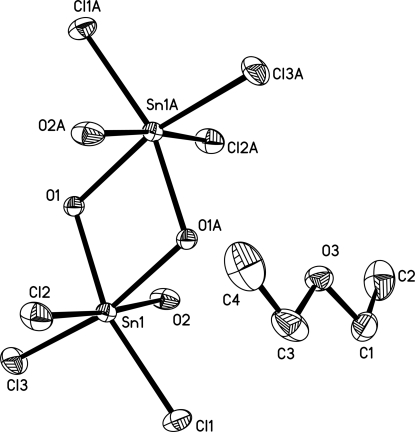
The molecular structure of (I), showing 50% probability displacement ellipsoids and the atom-numbering scheme. H atoms have been omitted for clarity.

**Fig. 2.**
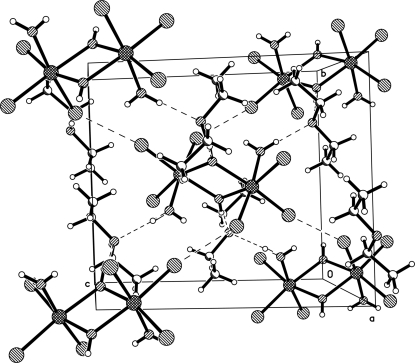
The infinite two-dimensional network structure of (I), H atoms have been omitted for clarity.

## supplementary crystallographic information

### Comment

We have synthesized the title compound unexpectedly, (I), and present its crystal structure here. The title compound consist of a centrosymmetric dimer (Fig. 1) in which the tin atoms have a distorted octahedral arrangement formed by three chlorine atoms, two hydroxy oxygen bridges and one water molecule. A further two water molecules are hydrogen-bonded to the hydroxyl oxygen atoms of the µ-OH bridges. The Sn—O distances in (I) (Table 1), are similar to those in related organotin carboxylates. The Sn—Cl bond lengths and the interbond angles lie within the ranges observed for other related complexes. The Sn1—O1 (2.072 (2) Å) and Sn1—O2 distance (2.183 (2) Å), (Table 1), are close to those reported for organotin carboxylates (Janas *et al.*, 1991).

### Experimental

The reaction was carried out under nitrogen atmosphere. 3-Thiophenemalonic acid (1 mmol) and sodium ethoxide (2.2 mmol) were added to the solution of benzene (30 ml) in a Schlenk flask and stirred for 0.5 h. Phenyltin trichloride (1 mmol) was then added to the reactor and the  mixture was stirred for 12 h at 338 K.The resulting clear solution was evaporated under vacuum. The product was crystallized from a mixture of diethylether/petroleum ether (1:1).Unexpectedly,a dimeric complex, was isolated from the filtrate. (yield 52%; m.p. 446 K). Analysis calculated (%) for C_4_H_13_Cl_3_O_3_Sn (Mr = 334.18): C,46.72; H, 4.49; O, 9.57. found: C, 46.52; H, 4.55; O, 9.62.

### Refinement

H atoms were positioned geometrically, with O—H = 0.85 and 0.93 Å and C—H = 0.96 and 0.97 Å for aromatic, methyl and methylene H atoms, respectively, and constrained to ride on their parent atoms, with *U*_iso_(H) = *xU*_eq_(C,*O*) where *x* = 1.5 for methyl H and *x* = 1.2 for all other H atoms.

### Crystal data

**Table:**

[Sn_2_Cl_6_(OH)_2_(H_2_O)_2_]·2C_4_H_10_O	*F*(000) = 648
*M**_r_* = 668.36	*D*_x_ = 1.999 Mg m^−^^3^
Monoclinic, *P*2_1_/*n*	Mo *K*α radiation, λ = 0.71073 Å
*a* = 10.1171 (15) Å	Cell parameters from 4261 reflections
*b* = 10.0212 (15) Å	θ = 2.4–28.2°
*c* = 11.2641 (18) Å	µ = 2.99 mm^−^^1^
β = 103.536 (1)°	*T* = 298 K
*V* = 1110.3 (3) Å^3^	Block, colorless
*Z* = 2	0.46 × 0.32 × 0.30 mm

### Data collection

**Table:**

Siemens SMART CCD area-detector diffractometer	1909 independent reflections
Radiation source: fine-focus sealed tube	1685 reflections with *I* > 2σ(*I*)
graphite	*R*_int_ = 0.027
φ and ω scans	θ_max_ = 25.0°, θ_min_ = 2.4°
Absorption correction: multi-scan (*SADABS*; Sheldrick, 1996)	*h* = −11→12
*T*_min_ = 0.340, *T*_max_ = 0.468	*k* = −11→11
5168 measured reflections	*l* = −7→13

### Refinement

**Table:**

Refinement on *F*^2^	Primary atom site location: structure-invariant direct methods
Least-squares matrix: full	Secondary atom site location: difference Fourier map
*R*[*F*^2^ > 2σ(*F*^2^)] = 0.023	Hydrogen site location: inferred from neighbouring sites
*wR*(*F*^2^) = 0.063	H-atom parameters constrained
*S* = 0.84	*w* = 1/[σ^2^(*F*_o_^2^) + (0.0489*P*)^2^ + 0.9726*P*] where *P* = (*F*_o_^2^ + 2*F*_c_^2^)/3
1909 reflections	(Δ/σ)_max_ = 0.001
102 parameters	Δρ_max_ = 1.05 e Å^−^^3^
0 restraints	Δρ_min_ = −0.68 e Å^−^^3^

### Special details

**Table:**

Geometry. All e.s.d.'s (except the e.s.d. in the dihedral angle between two l.s. planes) are estimated using the full covariance matrix. The cell e.s.d.'s are taken into account individually in the estimation of e.s.d.'s in distances, angles and torsion angles; correlations between e.s.d.'s in cell parameters are only used when they are defined by crystal symmetry. An approximate (isotropic) treatment of cell e.s.d.'s is used for estimating e.s.d.'s involving l.s. planes.
Refinement. Refinement of *F*^2^ against ALL reflections. The weighted *R*-factor *wR* and goodness of fit *S* are based on *F*^2^, conventional *R*-factors *R* are based on *F*, with *F* set to zero for negative *F*^2^. The threshold expression of *F*^2^ > σ(*F*^2^) is used only for calculating *R*-factors(gt) *etc*. and is not relevant to the choice of reflections for refinement. *R*-factors based on *F*^2^ are statistically about twice as large as those based on *F*, and *R*- factors based on ALL data will be even larger.

### Fractional atomic coordinates and isotropic or equivalent isotropic displacement parameters (Å^2^)

**Table:**

	*x*	*y*	*z*	*U*_iso_*/*U*_eq_	
Sn1	0.53184 (2)	0.46950 (2)	0.361848 (19)	0.02338 (10)	
Cl1	0.66543 (10)	0.58991 (10)	0.25532 (9)	0.0421 (2)	
Cl2	0.70089 (10)	0.30829 (10)	0.44565 (9)	0.0434 (2)	
Cl3	0.41097 (10)	0.33403 (10)	0.20324 (9)	0.0470 (2)	
O1	0.4121 (2)	0.4148 (2)	0.48000 (19)	0.0261 (5)	
H1	0.3447	0.3497	0.4651	0.031*	
O2	0.3762 (3)	0.6222 (3)	0.3036 (2)	0.0402 (6)	
H2D	0.3543	0.6636	0.2360	0.048*	
H2E	0.3425	0.6644	0.3549	0.048*	
O3	0.8141 (2)	0.7541 (2)	0.5809 (2)	0.0367 (6)	
C1	0.7894 (5)	0.8615 (4)	0.4926 (4)	0.0518 (11)	
H1A	0.7798	0.8260	0.4109	0.062*	
H1B	0.8653	0.9233	0.5091	0.062*	
C2	0.6621 (6)	0.9321 (5)	0.5016 (5)	0.0660 (14)	
H2A	0.5884	0.8694	0.4895	0.099*	
H2B	0.6415	1.0003	0.4402	0.099*	
H2C	0.6746	0.9720	0.5810	0.099*	
C3	0.9279 (4)	0.6715 (5)	0.5690 (4)	0.0537 (12)	
H3A	1.0075	0.7267	0.5730	0.064*	
H3B	0.9066	0.6263	0.4907	0.064*	
C4	0.9561 (5)	0.5717 (6)	0.6692 (5)	0.0720 (15)	
H4A	0.9715	0.6168	0.7464	0.108*	
H4B	1.0355	0.5210	0.6650	0.108*	
H4C	0.8797	0.5128	0.6610	0.108*	

### Atomic displacement parameters (Å^2^)

**Table:**

	*U*^11^	*U*^22^	*U*^33^	*U*^12^	*U*^13^	*U*^23^
Sn1	0.02419 (15)	0.02519 (15)	0.02104 (15)	0.00006 (8)	0.00583 (10)	−0.00080 (8)
Cl1	0.0446 (5)	0.0476 (5)	0.0398 (5)	−0.0069 (4)	0.0216 (4)	0.0052 (4)
Cl2	0.0438 (5)	0.0454 (5)	0.0423 (5)	0.0216 (4)	0.0127 (4)	0.0053 (4)
Cl3	0.0515 (6)	0.0523 (6)	0.0348 (5)	−0.0129 (5)	0.0053 (4)	−0.0163 (4)
O1	0.0273 (12)	0.0281 (12)	0.0234 (12)	−0.0074 (10)	0.0069 (9)	−0.0028 (9)
O2	0.0460 (15)	0.0483 (15)	0.0274 (13)	0.0203 (12)	0.0108 (11)	0.0104 (11)
O3	0.0334 (13)	0.0435 (14)	0.0355 (14)	−0.0047 (11)	0.0125 (11)	−0.0029 (11)
C1	0.071 (3)	0.047 (2)	0.037 (2)	−0.023 (2)	0.013 (2)	−0.0013 (19)
C2	0.076 (4)	0.048 (2)	0.063 (3)	0.002 (2)	−0.008 (3)	0.011 (2)
C3	0.034 (2)	0.074 (3)	0.056 (3)	−0.002 (2)	0.015 (2)	−0.025 (2)
C4	0.056 (3)	0.064 (3)	0.088 (4)	0.020 (3)	0.003 (3)	−0.012 (3)

### Geometric parameters (Å, °)

**Table:**

Sn1—O1	2.072 (2)	C1—C2	1.493 (7)
Sn1—O1^i^	2.090 (2)	C1—H1A	0.9700
Sn1—O2	2.183 (2)	C1—H1B	0.9700
Sn1—Cl1	2.3413 (9)	C2—H2A	0.9600
Sn1—Cl3	2.3469 (9)	C2—H2B	0.9600
Sn1—Cl2	2.3813 (9)	C2—H2C	0.9600
O1—Sn1^i^	2.090 (2)	C3—C4	1.485 (7)
O1—H1	0.9300	C3—H3A	0.9700
O2—H2D	0.8500	C3—H3B	0.9700
O2—H2E	0.8500	C4—H4A	0.9600
O3—C1	1.447 (5)	C4—H4B	0.9600
O3—C3	1.449 (5)	C4—H4C	0.9600
			
O1—Sn1—O1^i^	71.48 (9)	O3—C1—H1A	110.0
O1—Sn1—O2	83.66 (9)	C2—C1—H1A	110.0
O1^i^—Sn1—O2	84.28 (9)	O3—C1—H1B	110.0
O1—Sn1—Cl1	163.72 (7)	C2—C1—H1B	110.0
O1^i^—Sn1—Cl1	94.40 (6)	H1A—C1—H1B	108.4
O2—Sn1—Cl1	86.96 (7)	C1—C2—H2A	109.5
O1—Sn1—Cl3	93.27 (6)	C1—C2—H2B	109.5
O1^i^—Sn1—Cl3	163.56 (6)	H2A—C2—H2B	109.5
O2—Sn1—Cl3	88.04 (8)	C1—C2—H2C	109.5
Cl1—Sn1—Cl3	99.69 (4)	H2A—C2—H2C	109.5
O1—Sn1—Cl2	92.27 (7)	H2B—C2—H2C	109.5
O1^i^—Sn1—Cl2	90.68 (7)	O3—C3—C4	109.3 (4)
O2—Sn1—Cl2	174.32 (7)	O3—C3—H3A	109.8
Cl1—Sn1—Cl2	96.06 (4)	C4—C3—H3A	109.8
Cl3—Sn1—Cl2	96.16 (4)	O3—C3—H3B	109.8
Sn1—O1—Sn1^i^	108.52 (9)	C4—C3—H3B	109.8
Sn1—O1—H1	125.7	H3A—C3—H3B	108.3
Sn1^i^—O1—H1	125.7	C3—C4—H4A	109.5
Sn1—O2—H2D	129.1	C3—C4—H4B	109.5
Sn1—O2—H2E	121.4	H4A—C4—H4B	109.5
H2D—O2—H2E	107.7	C3—C4—H4C	109.5
C1—O3—C3	112.0 (3)	H4A—C4—H4C	109.5
O3—C1—C2	108.6 (3)	H4B—C4—H4C	109.5

Symmetry codes: (i) −*x*+1, −*y*+1, −*z*+1.

### Hydrogen-bond geometry (Å, °)

**Table:**

*D*—H···*A*	*D*—H	H···*A*	*D*···*A*	*D*—H···*A*
O1—H1···O3^i^	0.93	1.88	2.799 (3)	169.
O2—H2D···O3^ii^	0.85	1.89	2.736 (3)	176.
O2—H2E···Cl2^i^	0.85	2.40	3.179 (3)	152.

Symmetry codes: (i) −*x*+1, −*y*+1, −*z*+1; (ii) *x*−1/2, −*y*+3/2, *z*−1/2.
